# Guy's Hospital

**Published:** 1905-04-01

**Authors:** 


### The Hospital.

A Journal of

The Medical Sciences and Hospital Administration

Vol. XXXVIII.—No. 966. SATURDAY, APRIL 1, 1905.

### Guy's Hospital.

Mr. Cosmo Bonsor's appeal on behalf of Guy's Hospital, which we publish in another column, should certainly yield £100,000 during the current year, for two reasons especially. First, Mr. Bonsor is not making a fresh demand on behalf of Guy's, but merely asks the public to supply the balance of the funds sought to be raised in 1901. The money is required to defray the cost of renovations, buildings, and equipments which had become indispensable to the efficiency of the hospital, and to maintain more than 600 beds, being the whole number available for patients. Guy's may now be regarded as a model hospital well equipped and full of instruction to everybody who desires to see the most modern, economical, and efficient appliances and methods of treatment and administration. Probably in no single institution can all these matters be studied at greater advantage than they can be at Guy's to-day. Guy's now possesses complete accommodation for 227 nurses in the remarkable building known as the Henriette Raphael Nurses' Home; the additional operation theatres have the merit of being excellently arranged and equipped, at a cost so small as to place hospital committees generally under a debt of obligation to the governors for showing the way to avoid the mistake of providing a quarter of a mile, or so, of operation theatres, at a ruinous cost. The Light treatment installation has been cleverly organised, and the arrangements are so good as to be well worthy of the attention of hospital managers generally. The drainage has been reconstructed, the casualty department has been extended, open-air balconies have been added to all the medical and surgical wards, and an emergency ward for special casualty cases has now been provided. Further, and more important still, a central power station for the supply and distribution of light, heat, water, and power throughout the hospital buildings is now a feature of Guy's Hospital, and with the new laundry, capable of dealing with upwards of 40,000 articles per week, has had a material effect in reducing the cost per bed as shown in another column. The discussion which has taken place in regard to the advantages attaching to a hospital which has a medical superintendent in control of all the internal arrangements, as we show on page 12, is demonstrated at Guy's Hospital. The whole of the work has now been so systematised and brought into line that only ten porters are employed as against seventy-three at another metropolitan hospital, an immense saving, largely due to the introduction of successful mechanical methods in connection with the lifts, the telephone system, and other departments of work.

There are some hospitals, of which Guy's is one, which it is a pleasure to visit and study, because the greater the knowledge of the visitor in hospital matters the more he can appreciate its excellence, and the fact that there is always something to learn from such a visit, owing to the sound methods pursued and the amount of thought expended upon every detail of the organisation. Guy's Hospital, then, is entitled to its £100,000 on account of its merits, and from the fact that it constitutes an object-lesson to all who aim at securing the best, in connection with the particular hospital with which they may be associated. It follows that the comfort of all the patients in Guy's Hospital, due to the general *esprit* and the infinite care and supervision everywhere exercised, is probably unsurpassed.

Then, too, Mr. Bonsor's appeal commands public sympathy, because of the restraint and consideration for other hospitals, which have marked the whole conduct of the efforts of the Guy's Governors to raise money. They have deliberately refrained from appealing when other great hospitals were making demands upon the public purse. This fact has made Guy's one of the most popular of London hospitals with the more generous givers amongst us, and rightly so, for there is nothing more damaging to a voluntary institution than the indiscretion, which makes an over-zealous government so selfish as to be unable to appreciate the claims of other great institutions, which are equally dependent upon public support, of which each has a right to claim an adequate share.

Guy's men are known all over the world as the handy men of medicine. The training they receive and the discipline under which they acquire their knowledge are attended with equally good results to those secured by the Navy. Every Guy's man has a zealous affection for his Alma Mater, and we have little doubt that the result of the Guy's Shilling Fund will be remarkable in many ways. Administratively an example to many institutions, economically one of the most progressive of London hospitals, structurally as completely efficient as modern science and knowledge can make it in the circumstances, with a united government in which the medical staff are adequately represented, and where every official is contentedly working under a responsible chief, who unites and controls the whole establishment, Guy's Hospital is an institution, to which every thoughtful man and woman with money to spare may contribute liberally, with full confidence that the money will not be wasted. As generous donors they will directly aid one of the noblest hospitals in the world, and indirectly, by placing Guy's once and for all in adequate funds, they will render yet more efficient an institution, which, for educational purposes, may fairly be regarded as a power for good in the hospital world.

